# Foraminiferal isotopic evidence of abrupt mid-20th century onset of hydrographic instability in Nordic Seas inflow waters

**DOI:** 10.1038/s41598-025-32210-8

**Published:** 2025-12-16

**Authors:** Hans Petter Sejrup, Scott J. Lehman, Berit O. Hjelstuen, Lukas W. M. Becker, Rebekka H. Runarsdottir, Ulysses S. Ninnemann, Monica Ionita

**Affiliations:** 1 Department of Earth Science, University of Bergen, Bergen, Norway; 2 Institute of Arctic and Alpine Research, University of Colorado, Boulder, USA; 3 Rambøll Norge AS, Bergen, Norway; 4 Institute of Earth Science, University of Iceland, Reykjavík, Iceland; 5 Bjerknes Centre for Climate Research, Bergen, Norway; 6 Alfred Wegner Institute Helmholtz-Zentrum Centre for Polar and Marine Research, Bremerhaven, Germany; 7 Faculty of Forestry, Ştefan cel Mare University, Suceava, Romania

**Keywords:** Climate sciences, Ocean sciences

## Abstract

The flow of warm Atlantic waters into the Nordic Seas largely determines the transport of ocean heat to the Arctic and is a prominent feature of the Atlantic Meridional Overturning Circulation (AMOC). Here we provide a ~ 250-year-long (1750 to 1992 AD), annually- to sub-annually- resolved record of Nordic Seas inflow water characteristics inferred from changes in δ^18^O of planktic foraminiferal carbonate. The new record is reflective of upper ocean temperatures across the Atlantic Water Zone of the Nordic Seas and reveals a previously unrecognized increase in temperature instability ~ AD 1950 that appears to have impacted rates of Greenland Ice Sheet melting, Arctic sea-ice extent and Arctic Surface Air Temperature, delaying the regional response to Anthropogenic Global Warming by several decades. While the relationship between the sudden change in hydrographic conditions and AMOC strength and stability is not yet clear, the change in inflow characteristics ~ AD 1950 was clearly imprinted on deep waters overflowing the Nordic Seas Basin.

**Supplementary Information:**

The online version contains supplementary material available at 10.1038/s41598-025-32210-8.

## Introduction

**Fig. 1:**
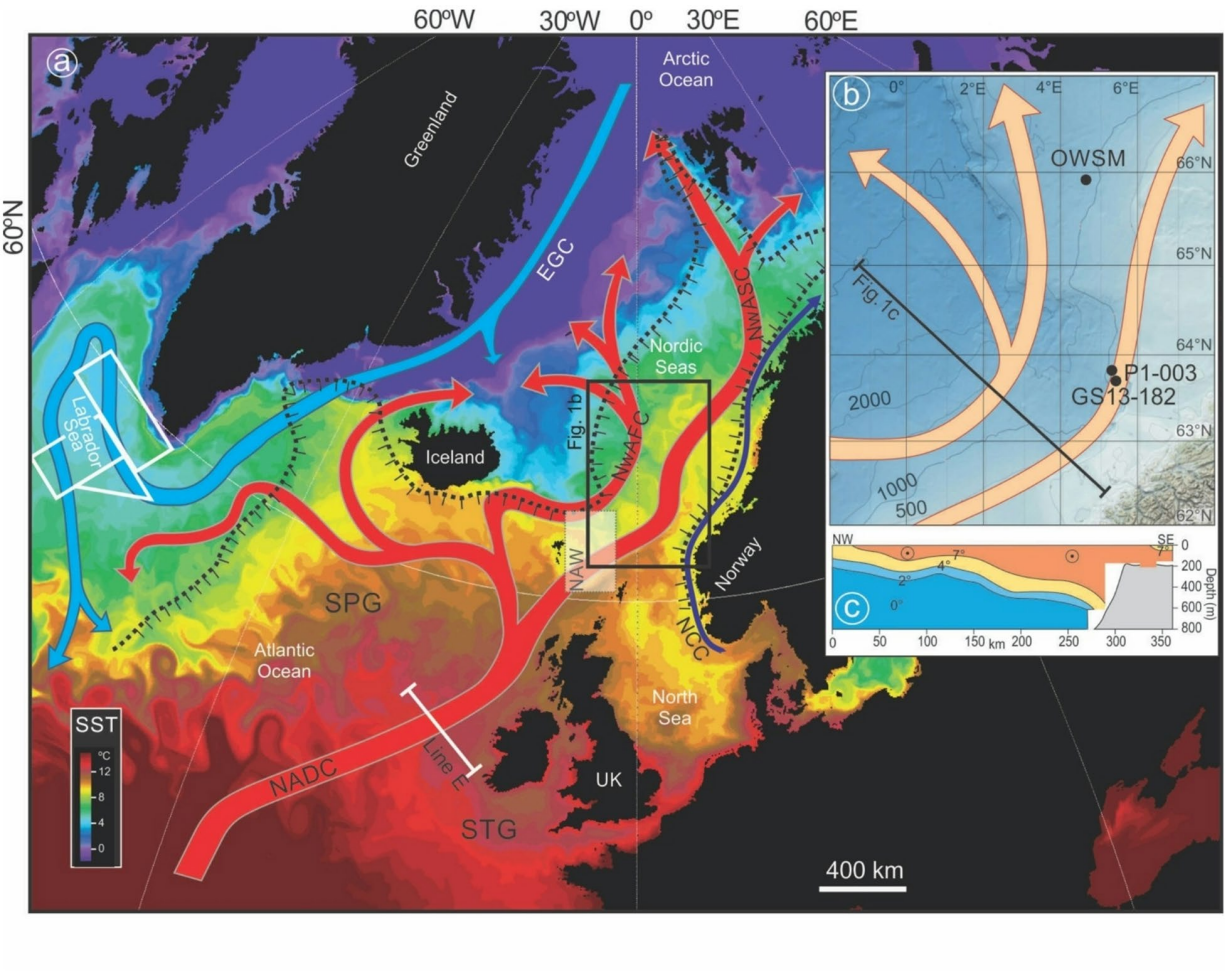
Location of oceanographic features and cores mentioned in text. (**A**) Main surface water flows of the northern North Atlantic and Nordic Seas Basin. The labels “EGC”,“NwAFC”, “NwASC”, “NADC” and “NCC” refer to the East Greenland, Norwegian Atlantic, Norwegian Atlantic Slope, North Atlantic Drift and Norwegian Coastal Currents, respectively (modified from Orvik and Niiler^67^ and Hansen and Østerhus^26^). “SPG” and “STG” refer to the Subpolar and Subtropical Gyres, respectively. The NAW box indicates the area of the reconstruction^43^ of the inflow water temperature variability (Fig. S5). Locations of insert map (**B**), “line E “ from Hatun et al.^17^ and of the Central Labrador Sea and West Greenland Current averaging domain from Reverdin et al.^48^ are also indicated. The Atlantic Water Zone of Carton et al.^37^ is defined by the 35 psu salinity contour within the Nordic Seas as indicated by the black dotted line. Background image courtesy of A.C. Coward (NOC, Southampton, showing mean early summer, daily mean sea-surface temperature). (**B**) Location of sediment cores GS13 and P1‐003, Ocean Weather Station Mike (OWSM^35^) and the location of the hydrographic profile^68^ in (**c**) on the Norwegian margin.

The region of warm Atlantic water inflow into the Nordic Seas Basin is a critical gateway within the climate system, accommodating the poleward transport of ocean heat to the Arctic Ocean and adjacent land and ice masses. These inflow waters are also a prominent surface expression of the so-called Atlantic Meridional Overturning Circulation (AMOC), which influences the coupled ocean and atmospheric transports of heat and freshwater at the global scale. However, the recent history of AMOC strength and associated changes in Meridional Ocean Heat Transport (MOHT) remain the subject of ongoing debate. Direct observations of diagnostic AMOC flows through instrumented ocean sections are available on a continuous basis only since 2004^1–3^. In addition, the physical and empirical basis underpinning various long-term estimates of AMOC strength, either from indirect hydrographic indicators during the instrumental period^4^ or related proxy indicators spanning earlier times^5–7^, has been called into question. This is in large part because indirect surface or near-surface hydrographic measures may be influenced by processes unrelated to meridional overturning^8,9^. And, more fundamentally, because the relative roles of deep convection in the Labrador, Irminger and adjacent Nordic Seas in driving AMOC variability remains unclear^10,11^. Most indirect measures rely on the presumed connection between AMOC strength and the hydrography and dynamics of the North Atlantic Subpolar Gyre (SPG, Fig. 1) which may influence negative buoyancy forcing and watermass conversion in the Labrador Sea region^12–14^, the development of basin-scale density differences needed to drive abyssal transport^15^, and/or the transport of relatively warm, salty subtropical waters to potential areas of deep water formation within and bordering the Nordic Seas^16,17^. In contrast to sources of decade- to century-scale AMOC variation seen in many numerical models, recent short-term observations of opposing upper ocean and deep ocean meridional flows in the northern North Atlantic are associated with surface-to-deep watermass conversion in and around the Irminger and Nordic Seas and not the northwestern North Atlantic, even at times of pronounced deep convection in the Labrador Sea^11^.

Here we report on a new ~ 250-year-long, annually- to sub-annually-resolved record of near-surface hydrography inferred from changes in δ^18^O of planktic foraminiferal carbonate in sediments retrieved from beneath the eastern branch of Atlantic water inflow to the Nordic Seas (Fig. 1). The gateway location and the combination of exceptional age control and temporal resolution permit us to evaluate changes in hydrographic coupling between the open North Atlantic and Nordic Seas not evident from either the paleoclimate or the instrumental records alone. This is in large part because the hydrographic record of the Nordic Seas prior to ~ AD 1949 is too fragmentary for meaningful synthesis while the temporal resolution of the many previous studies of late Holocene oceanography in the SPG and adjacent Nordic Seas^18–22^ has not been sufficient to detect hydrographic anomalies lasting just a few years.

## Results

### Geologic and hydrographic setting

Sediment core GS13-182-01CC (63° 38.643′ N; 5° 30.480′ E, Fig. 1), hereafter “GS13”, was raised from 960 m water depth on the Møre Margin, off Norway in the Storegga Slide Scar which was formed ~ 8200 yr BP and subsequently exposed to rapid along-slope sedimentation^23^. Sedimentary age control is provided by ^210^Pb/^137^Cs dating, identification of historical Icelandic tephra, ^14^C dating, and by correlation of high resolution (500 µm) XRF scanning records of Ca/Fe between core GS13 and nearby core P1-003^24,25^ (see Methods, Fig. S1 and Table S1). Isotopic measurements of the subpolar planktonic foraminifera *Neoglobquadrina incompta* (hereafter *N. inc.*) from the uppermost 2.5 m of core provide a temporal resolution ranging from 1.2 to 0.2 years for the period ~ AD 1750 to ~ AD 1992 with an estimated age uncertainty (1σ) ranging from 1 to 15 years, and 1 to 6 years for the period since the beginning of the instrumental record in AD 1870 (see Methods, Table S2 and S3). In addition, isotope analyses of different size fraction of foraminifera were performed on selected samples (see Methods, Table S4). Sampled sediments are uniformly fine-grained (≥ 98% less than 63 µm in size) with carbonate concentrations of just ~ 15–25% (Fig. S3). Planktonic foraminiferal assemblages were counted in the samples back to AD 1924 to assess faunal responses associated with isotope variability and to evaluate possible impacts of reworking (Supplementary Text, Fig. S2 and S4).

The core site lies beneath the main axis of warmest Atlantic water inflow to the Nordic Seas Basin, shown as the Norwegian Atlantic Slope Current (NwASC) in Fig. 1^26^. Any influence of the relatively fresh, cold Norwegian Coastal Current (NCC) to the east is restricted to the uppermost 10 m of the water column during June, July and August when the otherwise shelf-bound current may briefly extend seaward^27^. Waters of the NwASC represent a poleward extension of relatively warm, salty subtropical waters of the Subtropical Gyre (STG) that have been subject to variable mixing with colder, fresher waters of the SPG (Fig. 1). The SPG itself has varied significantly in extent and dynamic height over the instrumental period, influencing the hydrographic characteristics of NwASC via bathymetrically steered surface currents of the Rockall Trough and Faeroe Bank^17,28^. The nearest hydrographic station is Ocean Weather Station Mike (OWSM), located northwest of the core site and influenced by the waters of the Norwegian Sea Atlantic Front Current (NwAFC) (Fig. 1). Unlike the waters of the NwASC, Atlantic waters of the NwAFC have been modified by mixing with cold Polar waters to the west, within the Nordic Seas Basin. OWSM operated nearly continuously from 1948 to 2010, monitoring temperature and salinity from depths between 2000 m and the surface^27^.

### Comparison to the hydrographic record

**Fig. 2:**
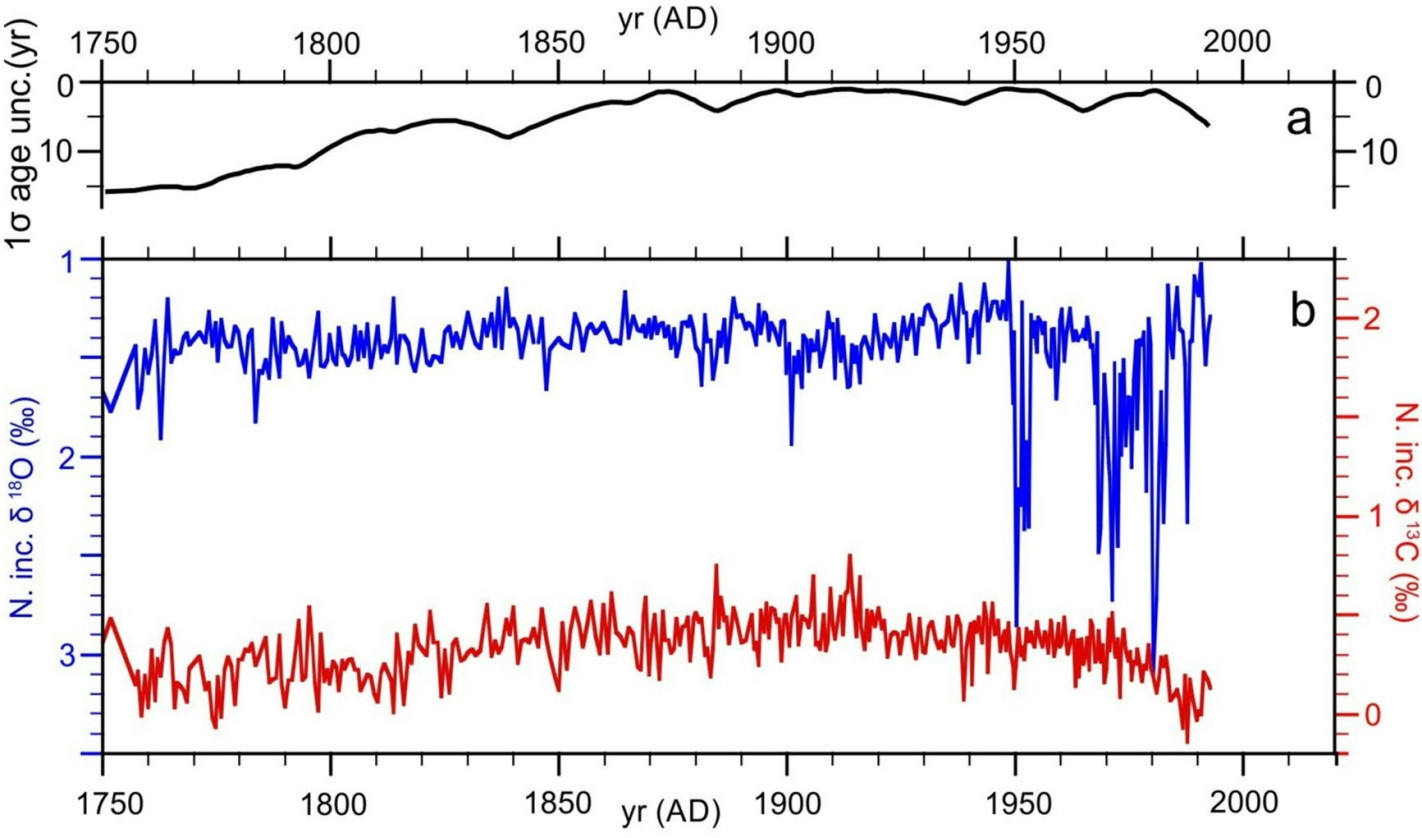
Timeseries of GS13 isotopic results. (**A**) Estimated chronologic uncertainty (1 σ) of the derived sediment age model. (**B**) Oxygen and carbon isotopic results for the planktonic foraminifera *N. inc.* shown on the derived age model. Core GS13 was raised from 960 m water depth on the Norwegian continental slope (see Fig. 1).

GS13 *N. inc.* isotopic results are shown in Fig. 2 on the derived age model along with an estimate of the time-varying age model uncertainty. δ^18^O varies coherently through a range of ~  ± 0.2 per mil for the period AD 1750–AD 1950 when an abrupt transition to much larger positive deviations of up to + 1.5 per mil occurs. Because of the unexpectedly large amplitude, positive δ^18^O anomalies were confirmed by multiple analyses and are presented as average replicate values (Table S3). Reworking of *N. inc.* from local glacial sediments is unlikely, since *N. inc.* are extremely rare in glacial age sediments in the region^29,30^ while both absolute and relative abundances of *N. inc.* within core GS13 are generally reduced during the interval marked by large δ^18^O variations and grain size distributions remain relatively constant (see Supplementary Text, Fig. S2 and Fig. S3). Reworking from areas presently influenced by colder waters such as the Iceland Plateau is also unlikely because the GS13 core site is situated under the easternmost branch of the inflow region marked by meridional currents and therefore relatively isolated from abyssal transport coming directly from the west (Fig. 1). In addition, associated δ^13^C results (Fig. 2) do not display anomalous excursions and are broadly consistent with expected late Holocene and post-industrial values in the eastern Norwegian Sea^31,32^. An increase in the δ^18^O variability of both *N. inc*. and in the planktonic foraminifer *Globigerina bulloides* post ~ 1940 AD was also observed in core P1-003^20,33^ (Fig. 1B), but the amplitude of variation was smaller, most likely as a result of reduced sampling resolution and lower sedimentation rate.

Based on previous isotopic studies it has been suggested that *N.inc*. in the region form their carbonate tests primarily during the spring and summer months and depths ranging from 10 to 100 meters^33,34^.

**Fig. 3:**
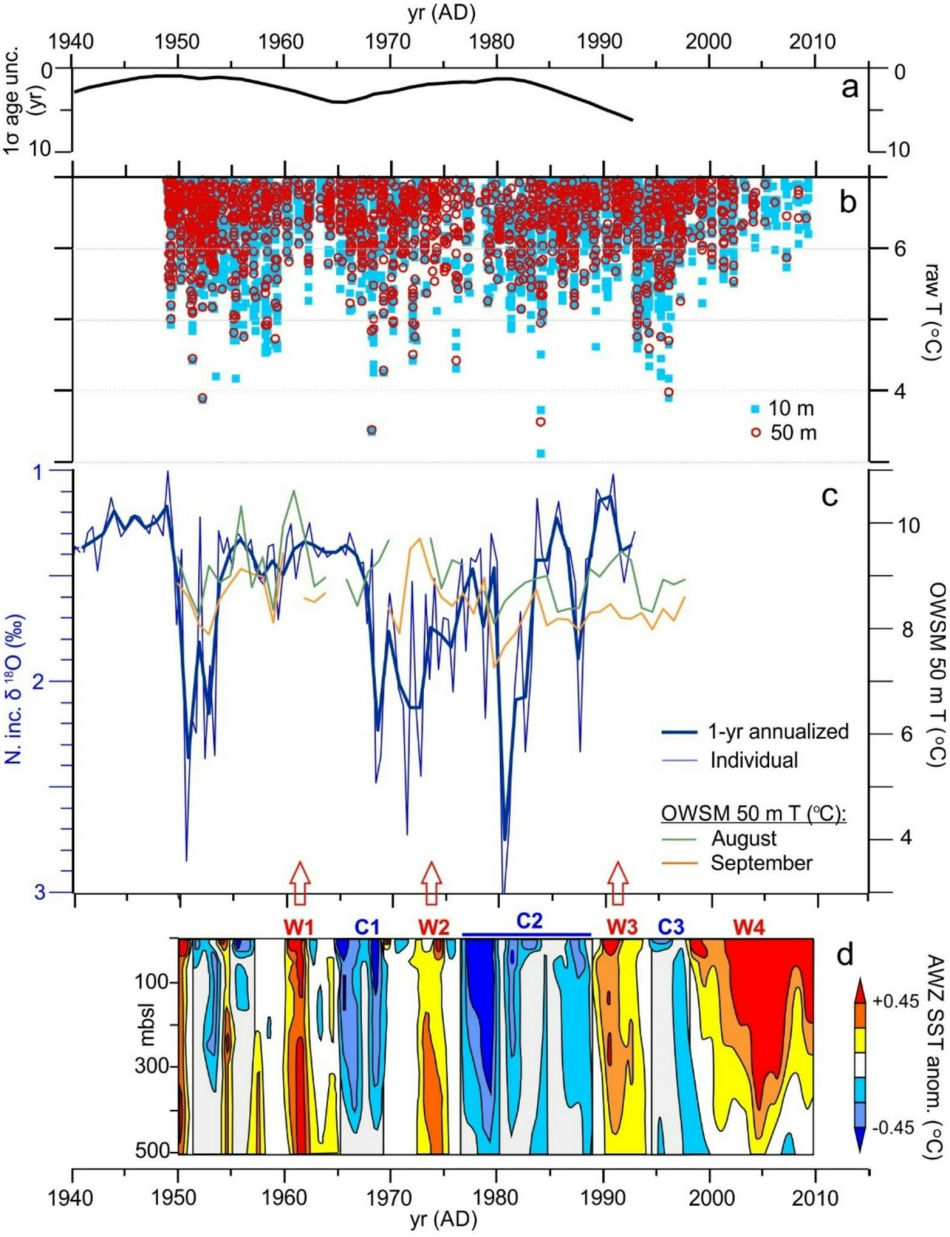
GS13 δ^18^O and Nordic Seas temperatures. (**A**) As in Fig. 2a. (**B**) Individual temperature observations less than 7 °C from 10 m (red) and 50 m (light blue) water depth at OWSM (see location in Fig. 1B). (**C**) Individual and annualized δ^18^O results from GS13 compared to observed variations of annual monthly-mean temperature at OWSM for August (green) and September (orange)^27,35^, scaled according to the temperature dependence of isotopic fractionation between sea water and calcite, as explained in the text. (**D**) Temperature variation of the upper 500 m of the water column in the Atlantic Water Zone (AWZ) within the Nordic Seas from Carton et al.^37^ along with previously defined cold (C1-C3) and warm (W1-W4) anomalies.

Comparison of post AD 1950 GS13 δ^18^O results to the instrumental temperature record at nearby OWSM^27,35^ for the presumed depth and season of *N. inc.* calcification^32,33^ show some common extrema, but correlations are low and not significant (R = 0.084 and 0.012 for August and September 50 m temperature vs. annualized δ^18^O, respectively) (Fig. 3). Further, scaling of the isotopic and temperature records according to the temperature dependence of δ^18^O of carbonate formed in equilibrium with seawater (~ 0.23 ‰/deg C^36^) implies isotopic temperature changes ~ 2–3 times larger than observed changes in monthly instrumental averages at OWSM. We note, however, that due to the sub-annual sampling resolution and lack of more precise knowledge of *N. inc.* habitat, individual (or, where relevant, replicate) GS13 δ^18^O measurements (thinner blue line, Fig. 3C) may represent hydrographic variation at sub-seasonal scales and at other depths and times of year, when individual OWSM temperature observations may vary by 3–4 °C (Fig. 3B). Accompanying changes in salinity (and water δ^18^O) may also influence the foraminiferal isotope composition, but the impact of this effect is expected to be less than a few tenths per mil (i.e., not more than ~ 1 °C isotopic temperature bias) based on observed temperature—salinity relationships at OWSM^20^. Changes in planktonic foraminiferal assemblages and abundance also indicate that variations in both plankton community structure and productivity occurred in association with the anomalies in the isotope record (Fig. S4), although it is not possible to conclude that these changes resulted directly from changes in in situ watermass temperature (See discussion in Supplementary Text).

We also compare the isotope record to a compilation of upper ocean temperature variation since AD 1950 averaged across the Atlantic Water Zone (AWZ) of the Nordic Seas Basin, previously defined as the area enclosed by the time mean 35 practical salinity contour at 100 m depth^37^ (Fig. 1A and 3c). The compilation is drawn largely from temperature and salinity profiles of the National Ocean Data Center’s World Ocean Data Base 2009^38^, and is presented as 1° × 1° monthly, 5-yr running mean temperature anomalies for standard ocean depth levels from 0 to 1000 m (i.e., 5 m, 25 m, or 50 m increments that increase with depth). Departures from depth-level mean temperatures are largely coherent to a depth of 400 m or more, with warm and cold anomalies ranging in duration from ~ 5–15 yr and previously identified as warm and cold intervals W1-W4 and C1-C3, respectively^37^. W3, early and late C2, and C1 all appear to have counterparts in the isotope record, along with the warm to cold transition shortly after 1950 AD. Both the AWZ compilation and the isotope record indicate a common interval of coldest temperatures (highest δ^18^O) occurring at or shortly before AD 1980. In addition, the contrast between temperature extrema of C2 and W3 in the regional compilation is greater than at OWSM, with a relative amplitude that is more consistent with the rebound to lower isotopic values in the sediment record. Differences in timing of AWZ event boundaries and their suggested equivalents in the sediment record most likely result from uncertainties in the sediment age model, which reach local maxima around the time of the W1/C1 transition (the mid-1960’s) and during late C2 (the mid-1980’s, near the sediment core top).

**Fig. 4:**
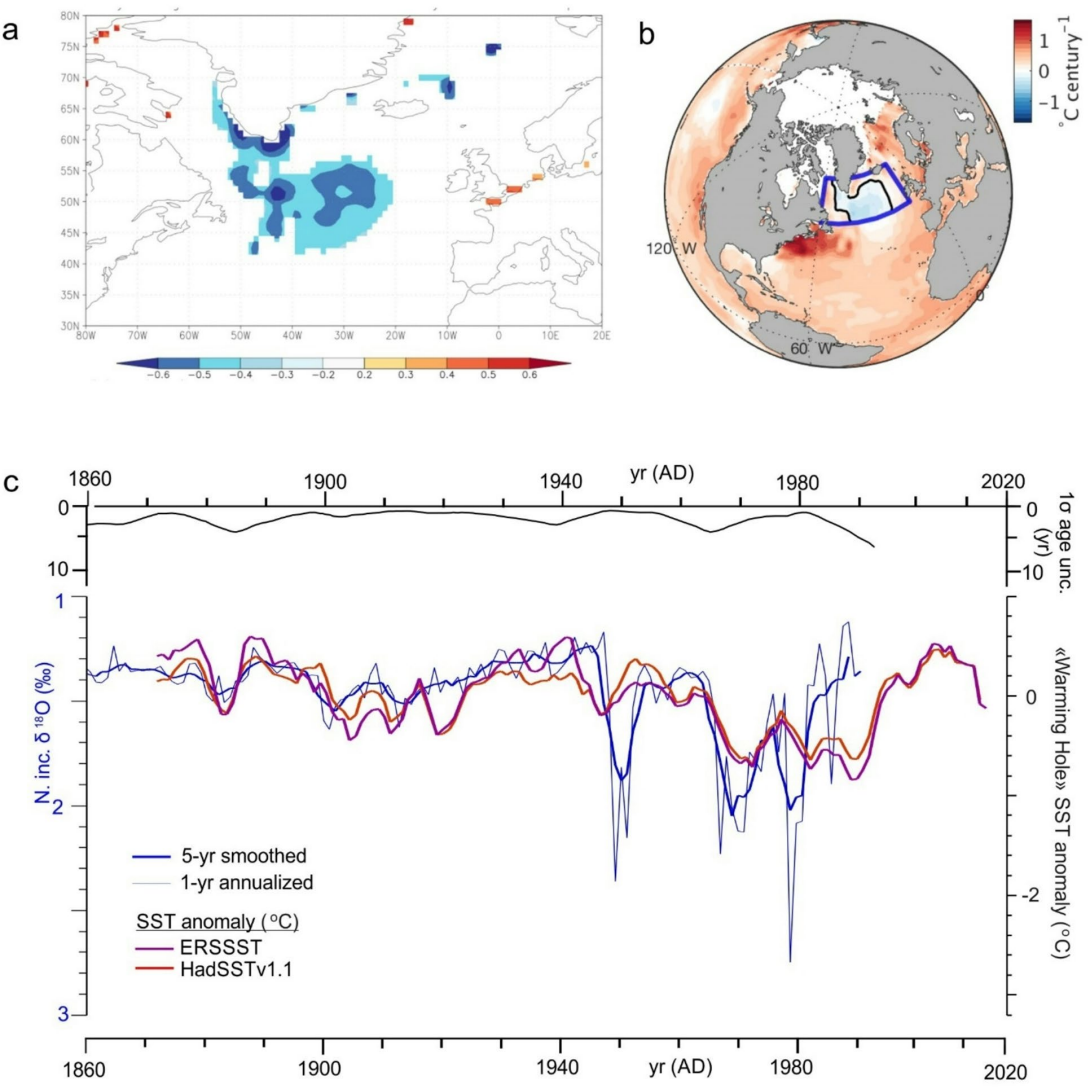
GS13 and SPG temperatures. (**A**) Point correlation of the GS13 δ^18^O and annual mean gridded HadlSST1^40^ reconstruction since 1870 AD at a lag of 2 yr (i.e., GS13 δ^18^O follows upstream North Atlantic temperature by 2 yr). Negative correlations are positive in isotopic temperature and correlations are relatively stable for lags of 2 to 7 years. (**B**) Distribution of linear trends in the HadlSSTv1.1 data showing the location of persistent cooling defining the so-called North Atlantic “warming hole”^41^. (**C**) SST series extracted over the warming hole domain (blue box in **B**) for both HadlSST and ERSSST gridded data sets from Osman et al.^41^ compared to the GS13 δ^18^O series for the instrumental period beginning 1870 AD.

Composite temperature variations of the Nordic Seas Basin AWZ are widely attributed to combined changes in the temperature and volume transport of waters coming from the North Atlantic SPG and STG mixing region^28,37,39^. We therefore performed a grid point correlation of the GS13 δ^18^O series to the HadlSSTv1.1 gridded Sea Surface Temperature (SST) data set available for the North Atlantic since AD 1870^40^. Strongest correlations are obtained at 2-yr lag (δ^18^O follows SST) with a well-defined “footprint” extending from the south coast of Greenland and into central SPG (Fig. 4A), with negative correlation corresponding to a positive relationship between isotopic paleo-temperature and SST. The region of strong correlation is readily recognizable as coincident with the so-called North Atlantic “warming hole”, previously defined as the region of persistent, anomalous negative SST trend within the North Atlantic basin^6^ (Fig. 4B). Time-series of “warming hole” SST derived by Osman et al.^41^ for both the HadlSST^40^ and ERSSST^42^ data sets and the GS13 δ^18^O series (Fig. 4C) show significant correlation (R = 0.55 and 0.66 for 5-yr smoothed δ^18^O vs. HadlSST and ERSSST respectively, P < 0.01, reduced DOF = 28). Correlations are larger (R = 0.65 and 0.71, resp., P < 0.01, reduced DOF = 22) when the isotopic results after 1982 AD are excluded, likely due to increasing chronological uncertainty near the sediment core top (Fig. 4C).

Although the amplitude of δ^18^O variation in core GS13 after ~ AD 1950 remains surprising, relationships above indicate that the timing and relative amplitude of isotopic variation are consistent with the history of observed mean temperature variation within the Nordic AWZ and the SPG (i.e., Figs. 3 and 4, respectively), which is the likely upstream source of AWZ variability itself^37^. Various studies^17,28^ suggest increased routing of SPG waters across Rockall Trough and more or less directly into the NwASC (i.e. through line “E” of Hatun et al.^17^, shown in Fig. 1A) at times when the SPG is colder and more extensive. This may explain some of the difference between estimated “isotopic temperature” variation at the core site and observed temperature variation at OWSM (Fig. 3B); i.e., we can expect the amplitude of temperature response to addition of relatively cold SPG waters to be larger in the NwASC than in the neighboring NwAFC (and at the location of OWSM) because the Atlantic waters of the NwAFC have already been modified by mixing with the cold, fresh Polar waters within the Norwegian Sea (c.f. Figure 1A) and no longer represent a warm end member for the basin. In addition, temperature measurements in the core of the NAW (North Atlantic Water) directly upstream from GS13 in the Faroe-Shetland region^43^ show variability of up to c. 2.5 °C in annualized data for the period between 1950 and 2005 (Fig. S5). An alternative explanation involving lateral transport of living foraminifera that calcified largely in the SPG or in SPG-spawned cold core rings and subsequent deposition at the core site is unlikely to have contributed significantly to the observed isotope record since *N. inc.* are native to the southeastern Norwegian Sea, dominating core top assemblages in the region^44^ (Supplementary Text, Fig. S2).

**Fig. 5:**
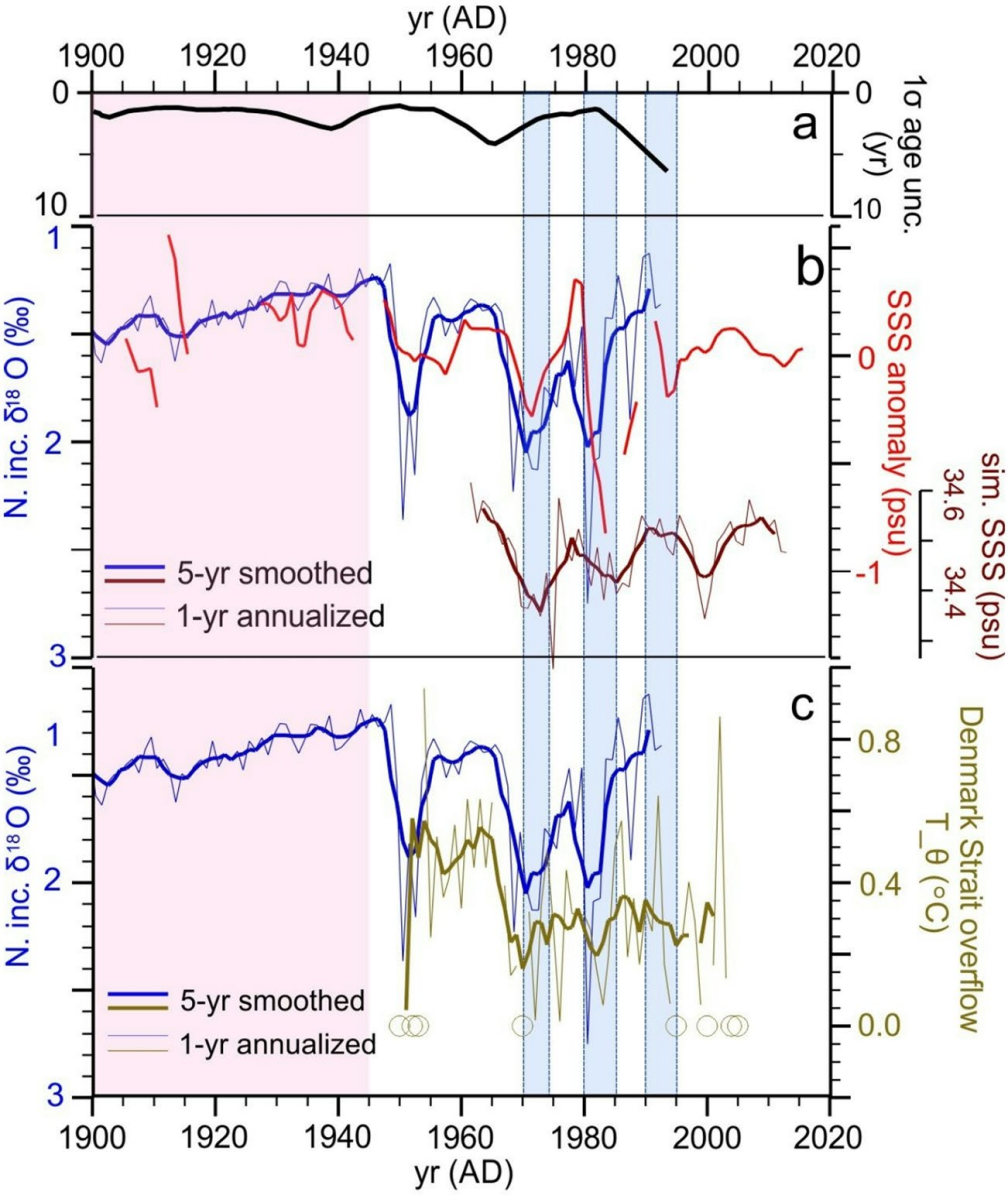
GS13 δ^18^O, Labrador Sea, Denmark Strait temperatures and salinities. (**A**) As in Figs. 2–4. (**B**) 1- and 5-yr smoothed GS13 δ^18^O (blue lines) and Central Labrador Sea instrumental SSS from the compilation of Reverdin et al.^48^ (red line) and Labrador Sea SSS as simulated in response to “Greenland/Arctic” atmospheric blocking from Ionita et al.^60^ (brown lines). (**C**) GS13 δ^18^O (as in **B**) along with annual mean potential temperature of Denmark Strait overflow waters since 1949 AD after Eldevik et al.^43^. Open circles denote missing years in the instrumental record. Blue horizontal bars indicate the timing of the Great Salinity Anomalies after Belkin^45^. Red/white transition in background color indicates the start of pronounced instability in hydrographic characteristics of Nordic Seas inflow waters.

Isotopic variations in core GS13 also show similar timing and relative amplitude to variations of annual mean potential temperature of deep overflow waters leaving the Nordic Seas Basin through Denmark Strait (Fig. 5C) for the period of Denmark Strait observations beginning in AD 1949^43^. Correlation of the complete 5-yr smoothed records is R = 0.57, although the significance of the correlation is limited (P = 0.11 for reduced DOF = 9). The correlation increases to R = 0.69 when excluding the period after AD 1982 (P = 0.09, reduced DOF = 7) when the dating uncertainty is larger. These relationships are consistent with prior analysis indicating that hydrographic characteristics of surface and near-surface inflow waters are imparted to overflow waters by recirculation and convection within the Nordic Seas Basin in just a few years^43^. While chronological uncertainties prohibit a direct evaluation of the phase of the relationship between the isotopic record and the relatively brief instrumental record of Denmark Strait overflow temperature, the instrumental record itself indicates that shared temperature and salinity signals in Atlantic inflow waters and recirculated Atlantic waters within the Nordic Seas Basin lead the related Denmark Strait overflow signal^43^.

Taken together, covariation of the GS13 δ^18^O series and instrumental records of hydrographic variation within the SPG, the Nordic Seas Basin AWZ and Denmark Strait overflow waters suggest the isotopic record may be used to track the hydrographic coupling between the open North Atlantic and the Nordic Seas Basin prior to the period when more extensive and better-synthesized surface and subsurface observations within the Nordic Seas Basin became available ~ AD 1949 (Table S5). By chance, this change in hydrographic coverage appears to coincide with a significant increase in the amplitude of δ ^18^O response in core GS13 to SST variability within the SPG ~ 1950 AD (Fig. 4C), which may explain why this seemingly marked transition in hydrographic coupling between the two regions has not been noted previously. Within the SPG itself, the shift at ~ AD 1950 is marked by a transition from a sustained interval of rising but variable temperatures to one of overall cooling that continued until the early 1990’s (Fig. 4C). A similar transition has been noted previously in paleo-temperature records of the Iceland Basin, in the SPG-STG mixing region^21^.

## Discussion

Of the several large isotopic anomalies seen in core GS13 after ~ AD 1950, maxima at ~ AD 1970 and ~ AD 1982 undoubtedly record the so-called Great Salinity Anomalies beginning around those times^45^ (Fig. 5). These events are detected initially as anomalously low sea surface salinity (SSS) within the West Greenland Current^46,47^ and then propagated through the cyclonic SPG circulation and onward to the Nordic Seas. The imprint on the inflow waters is here indicated by the similarity of GS13 δ^18^O and the record of SSS in the West Greenland Current and adjacent Central Labrador Sea^48^ (Fig. 5A and B). Given the strong association between SSS and SST within the SPG, the isotopic response in near-surface foraminiferal carbonate at the location of GS13 must be dominated overwhelmingly by the associated low SST signal.

**Fig. 6:**
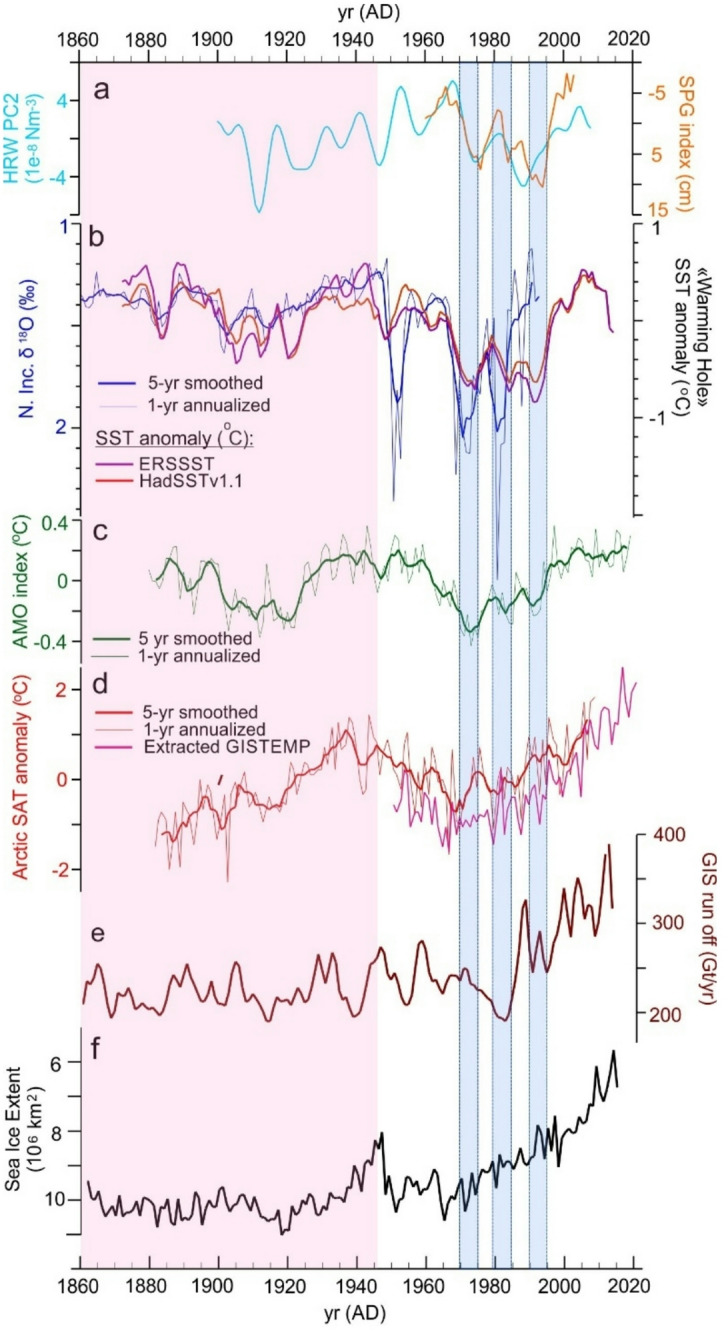
Observed and simulated records of North Atlantic and Arctic climate variables discussed in the text. (**A**) Wind Stress Curl PC2 (blue line) of Hakkinen et al.^53^ and the simulated SPG Index (red line) of Haatun et al.^17^. (**B**) GS13 δ^18^O and SPG “Warming Hole” SST, as in Fig. 4. (**C**) 1-yr annualized and 5-yr smoothed AMO index^69^, https://psl.noaa.gov/data/timeseries/AMO/ (accessed 6/2022). (**D**) 1-yr annualized and 5-year smoothed Arctic SAT anomaly compiled by Chylek et al*.*^56^ (red lines) and extracted from GISTEMP^70^ for “Arctic Ocean and Land” by Rantanen et al*.*^71^ (pink line). (**E**) Reconstructed GIS runoff of Trusel et al.^58^. (**F**) Summer (JAS) Arctic sea-ice extent from Walsh et al.^57^. Horizontal blue bars and background color as in Fig. 5.

Covarying multi-decadal trends of SPG SST and GS13 δ^18^O (Fig. 4C) are similar to N. Atlantic-wide SST anomalies of the Atlantic Multi-decadal Oscillation (AMO^49^, more recently described as Atlantic Multi-decadal Variability or AMV, Fig. 6), as might be expected given the spatial overlap amongst the three measures and their shared responses to a combination of unforced internal variation of the ocean–atmosphere system and, particularly after ~ AD 1980, Anthropogenic Global Warming (AGW)^50–52^. The instrumental record of the AMO is also well explained by variations of the regional atmospheric forcing. As demonstrated by Hakkinen et al.^53^ changes in the frequency of winter-time atmospheric blocking events over the North Atlantic and Western Europe influence both the strength of the North Atlantic gyres and the regional ocean–atmosphere heat exchange, in large part through associated changes in the strength and pattern of the surface wind forcing. These give rise to two distinct patterns of anomalous wind stress curl; the first associated with the SPG-STG inter-gyre region (and well correlated with the North Atlantic Oscillation^54^), and the second, with the strength of the individual wind-driven gyre circulations. Time-series of the second mode (wind stress curl PC2 of Hakkinen et al.^53^) appear to explain the multi-decadal trends and decadal variability evident in both the SPG SST and GS13 δ^18^O series and is also coherent with anomalous dynamic height of the SPG (the Subpolar Gyre Index or SPGI of Hatun et al.^17^ (Fig. 6A and B)). The SPGI reflects the strength and extent of the SPG^55^, such that positive anomalies of dynamic height are associated with cooling and expansion of the SPG leading to anomalous intrusion of relatively cold, fresh SPG waters into the NAC and the Nordic Seas inflow (Fig. 1). Conversely, at low index state, entrainment of SPG waters into the NAC is restricted, permitting greater STG influence and higher temperature and salinity of inflow waters^16,17^.

The AMO is also well correlated with the instrumental record of Arctic-wide Surface Air Temperature (SAT)^56^ and appears to explain first order changes in Arctic sea-ice extent^57^, including an interval of sustained warming and sea-ice loss during the first half of the 20th Century that occurred prior to the most pronounced AGW^53^ (Fig. 6). The GS13 isotopic record suggests that this interval ended not gradually, but in an abrupt transition to a period of increased hydrographic instability in Nordic Seas inflow waters, a large and abrupt expansion of Arctic sea ice that reversed nearly a half-century of progressive retreat along with transient reductions of Greenland Ice Sheet (GIS) melt rate (Fig. 6A and F)^58^. Indeed, it appears that the hydrographic changes indicated by the GS13 δ^18^O record may have helped to delay the regional cryospheric response to AGW, which does not appear to dominate the regional signal again until after ~ AD 1980. In particular, while both the GS13 isotopic record and Atlantic-wide AMO Index appear to correspond with transient coolings seen in the instrumental record of Arctic SAT, transient reductions in temperature of poleward flowing inflow waters implied by the isotope record appear to correspond with discrete decade-scale reductions in simulated GIS melt rate. While the relationship between SPG SST, Arctic sea ice extent, and GIS melt rate and Arctic paleo-temperature (i.e. Arctic2k^59^) has been noted previously^58^, we caution that the previously observed relationship between simulated GIS melt rate and the Arctic2k temperature reconstruction may arise in part because the latter includes multiple paleo-temperature records obtained from GIS ice cores.

Another atmospheric blocking pattern extending from Greenland towards Northern Scandinavia is associated with anomalous wind stress east of Greenland and over the Arctic Ocean Basin, and may help to explain the remote influence of Arctic Ocean sea-ice on the temperature and salinity of the SPG^57,60^. The mechanism involves the anomalous convergence of sea-ice north of Greenland and subsequent southward transport through Denmark Strait, the West Greenland Current and into the Labrador Sea, where the associated low salinity, low temperature anomaly can be entrained by the SPG and returned to the Nordic Seas inflow region^61,62^. Ionita et al.^60^ previously used a finite-element ocean/sea-ice model forced by re-analyzed atmospheric pressure fields for the period 1948 – 2010 (with results for 1960–2000) to represent the development of the GSA of the 1970’s and its possible impact on the AMOC^62,63^. Here we note that the simulated mean surface salinity of the Labrador Sea associated with this “Greenland/Arctic” blocking pattern^60^ also appears to represent observed SSS in Central Labrador Sea^48^ and the development of isotopic anomalies we observe in the Nordic Seas inflow region (Fig. 5B).

SPG state^17,55,64^ simulated changes of Central Labrador Sea SSS^60^ are each associated with changes in AMOC strength within their respective model frameworks, as are the windstress-modulated poleward transports of warm, salty STG water^53^, SPG “Warming Hole” SST^5,6^ and AMO Index state^65^ (Fig. 1). The covariation of these indices with the record of δ^18^O in core GS13 might therefore suggest that the latter provides a well-resolved proxy of relative AMOC strength since AD 1750. However, as noted in the Introduction, considerable uncertainty remains regarding individual model representations of AMOC forcing and response and the reliability of many indirect surface and near-surface measures of AMOC strength have been called into question. Instead, we emphasize our observation that the GS13 δ^18^O record exhibits robust correlations to instrumental records of SPG SST (AD 1870 to 1987) (Fig. 4) and the temperature of deep waters overflowing Denmark Strait (AD 1949 to at least 1982) (Fig. 5), suggesting that variations in dynamics of the SPG may have influenced surface to deep water conversion in the Nordic Seas and not only in the open Labrador Sea as seen in some numerical models. Further, the GS13 δ^18^O record indicates that the impact of SPG surface hydrographic variation on Nordic Seas inflow waters changed dramatically within a few years of AD 1950, suggesting that water mass conversion in and around the Nordic Seas may have become vulnerable to anomalous hydrographic forcing at that time. The GS13 δ^18^O record also appears to explain some sub-decadal variability of Arctic SAT and GIS melt rate records not predicted by the Atlantic-wide AMO Index, suggesting that the new record may provide a fuller depiction of relative changes in MOHT to the Arctic Ocean and surrounding ice and land masses.

We present a new ~ 250-year-long, annually- to sub-annually-resolved record of near-surface hydrography inferred from changes in δ^18^O of planktic foraminiferal carbonate in sediments retrieved from beneath the warm, eastern branch of Atlantic water inflow to the Nordic Seas. The record shares many characteristics of the instrumental record of upper-ocean temperature averaged across the so-called Atlantic Water Zone of the Nordic Seas Basin available since AD 1949 and is also well correlated with the history of SST and SSS within the SPG since AD 1870, permitting an analysis of the coupling between the open North Atlantic and Nordic Seas over the last two-and-a-half centuries. The influence of the SPG on the temperature of Nordic Sea inflow waters appears to have increased dramatically within a few years of AD 1950. SPG-sourced variability at this time includes strong responses to the Great Salinity Anomalies of the 1970’s and early 1980’s. The sudden change in inflow characteristics beginning ~ AD 1950 appears to have been imparted to deep waters overflowing Denmark Strait within just a few years, suggesting that the SPG has influenced surface- to deep-water mass conversion in or around the Nordic Seas and not (or, not only) in the open NW Atlantic. Abrupt changes in warm water inflow implied by the new record also appear to have had a significant influence on rates of GIS melting, Arctic sea-ice extent and Arctic SAT, delaying the regional cryospheric response to AGW by several decades.

The overall record can be well-explained by previously reconstructed changes in the frequency of atmospheric blocking and their influence on the pattern and strength of wind stress forcing—the same forces that create the North Atlantic gyres in the first place^53^. Although the new record is well correlated with a number of indirect measures of AMOC strength, the true impact of the increase in hydrographic instability of Nordic Seas inflow waters ~ AD 1950 on the strength of the large-scale overturning circulation remains unknown. It is also noteworthy that this apparently sudden change in the SPG influence on the Nordic Seas occurred just as hydrographic observations in the Nordic Seas became extensive enough to permit meaningful synthesis, which may explain why this abrupt and seemingly unprecedented transition has not been documented previously.

## Methods

### Sampling and analysis

Adjacent 0.5-cm-thick samples (~ 13 cm^3^) were taken continuously for the investigated part of the core. Samples were treated with 35% hydrogen peroxide (H_2_O_2_), shaken for 48 h at 150 rpm and subsequently wet sieved with a sieve mesh size of 63, 125, 150 and 1000 μm (Fig. S3). The 150 to 1000 μm fraction was used for preliminary faunal analysis (see Supplementary Text, Fig. S4). Isotopic analyses were performed on 5 to 10 specimens of *N. inc.* picked from the 125–1000 μm fraction of 375 individual stratigraphic levels. Measurements were performed at the Facility for advanced isotopic research and monitoring of weather, climate and biogeochemical cycling (FARLAB) at the Department of Earth Science, University of Bergen, with an external precision of ≤ 0.08‰ for δ^18^O based on long term replication of in house standard CM12. Replicate analyses of *N. inc*. from eight different levels were obtained in order to confirm unexpectedly high δ^18^O values and are presented as replicate mean values (Table S3, Fig. 2). Mono-specific analyses were carried out on eight selected levels on the fractions 150–212 and 212–250 μm to investigate the effect of size fraction. These analyses rule out size influences as a potential source of large variability (Table S4).

### Chronology and statistical analyses

The sediment chronology and derived age uncertainties are from Becker et al.^24^ and summarized in the Fig. S1. Once transferred to the derived age model, the isotope record was annualized (or interpolated) to a 1-yr time step using the Analyserie program^66^ and, where appropriate, smoothed (5-yr) for comparison to the instrumental record (Fig. 3). Spatial correlations (Fig. 4) were generated using the KNMI climate explorer (https://climexp.knmi.nl/).

## Supplementary Information

Below is the link to the electronic supplementary material.

Supplementary Material 1

## Data Availability

The oxygen and carbon isotope data from core GS13 is available at Zenodo (10.5281/zenodo.10617999).
