# Supplementary material for: Foraminiferal isotopic evidence of abrupt mid-20th century onset of hydrographic instability in Nordic Seas inflow waters
Source: Sci Rep. 2025 Dec 16;16:2483. doi: 10.1038/s41598-025-32210-8 (PMC12820267; doi:10.1038/s41598-025-32210-8)
Supplement: Supplementary file 1 — Supplementary Material 1 [file 41598_2025_32210_MOESM1_ESM.docx]

Supplementary Materials for

**Foraminiferal isotopic evidence of abrupt mid-20th Century Onset of Hydrographic Instability in Nordic Seas Inflow Waters**

Hans Petter Sejrup *et al.*

*Corresponding author. Email: [sejrup@uib.no](mailto:sejrup@uib.no)

**This PDF file includes:**

Supplementary Text

Figures S1 to S4

Tables S1 to S5

Supplementary Text

**Reworking and redeposition** As noted in the main text, re-working of *N. inc.* from glacial age sediments or from colder locations is highly unlikely. Firstly, absolute abundances of *N. inc*. show reductions during the periods of anomalously elevated δ^18^O, opposite to what would be expected by reworking and/or transport of exogenous specimens of *N. inc.* (**Fig. S2a, b**). Further, the relative abundance of *N. inc*. is generally consistent with the temperature interpretation of overall δ^18^O record, indicating warming conditions prior to AD 1950, followed by the onset of cooling at that time, and then a temperature recovery beginning in the 1980’s, consistent with hydrographic data discussed in the main text. The general low absolute and relative numbers of *Neogloboquadrina pachyderma* (**Fig. S2a**, **b**) indicate that reworking from glacial sediments in the region is unlikely as these sediments are dominated by this species ^1,2^. Further, there is no evidence in sediment grain size data (**Fig. S3**) for sediment disturbance that might be expected in response to episodic “inworking” of exogenous material at times of anomalously elevated δ^18^O.

**Chemical effects** Previous work on GS13 sediments ^3^ notes excellent preservation of biogenic carbonate in the form of both foraminifera and coccolithophorids (with the latter greatly dominating the carbonate fraction). Thus, dissolution is not expected to have significantly impacted on the measured δ^18^O values. Further, photomicrographs of individual foraminifera do not show signs of secondary or authigenic carbonate formation ^3^. And while authigenic carbonate formation may occur without leaving substantial visual evidence (see for example Pearson *et al*.^4^), we note that authigenic formation would be extremely unlikely within the Storegga Slide Scar, where deposition rates of silt and clay are extremely high, carbonate concentrations are never greater than 25% dry weight, and foraminiferal concentrations average just 64 specimens per gram ^3^. Indeed, such high levels of sedimentary non-carbonate silt and clay are generally considered to be protective, greatly mitigating against chemical recrystallization.

**Planktonic foraminiferal micropaleontology** Planktonic foraminiferal assemblages have so far been counted in the fraction >150 from 141 samples back to 1924 AD and eight species were routinely identified. As the water content are fairly constant at c. 60% and the rate of sedimentation relatively uniform at c. 0.9 cm/year, we consider the number of planktonic foraminifera pr gram (**Fig. S4**) to reflect variability in the flux. The number per g show a general similarity to studies on primary productivity in the region with a strong decline after c. 1940 AD ^3,5,6^ . It also seems also to be a strong reflection of the isotope anomalies and the number of planktonic foraminifera pr. g (Fig. S4). The assemblages in G13 are generally dominated by *N. inc.* with *T. quinqueloba* and *G.bulloides* as important secondary species (**Fig. S4**). This is in good agreement with previous work on late Holocene assemblages from the region ^2,7-9^. More southerly distributed species like *G. inflata*, *G. glutinata* and *O. universa* have been considered to reflect strength of Atlantic water influence into the Norwegian Sea ^10^. Especially the percent of the deep dwelling and winter and spring crystallizing *G. inflata* ^11^has a decline during the strongest isotope anomalies. The distribution of *T. quinqueloba* is interesting since it generally shows an increase at the onset of the period with the anomalies, however during the first part of the strongest anomalies it decrease to zero percent. An increase in this species may be taken as an evidence of a general cooling, according to its present day distribution ^12^. However, the variability in the distribution of *T. quinqueloba* may also reflect the relationship between this species and productivity ^13^, which seems to decline at the level of the anomalies.

Taken together, these relationships support our conclusion that our isotope data reflect large scale oceanographic variability starting close to 1950AD. How large part of this variability can be attributed to temperature conditions can, however, not be determined from these data.


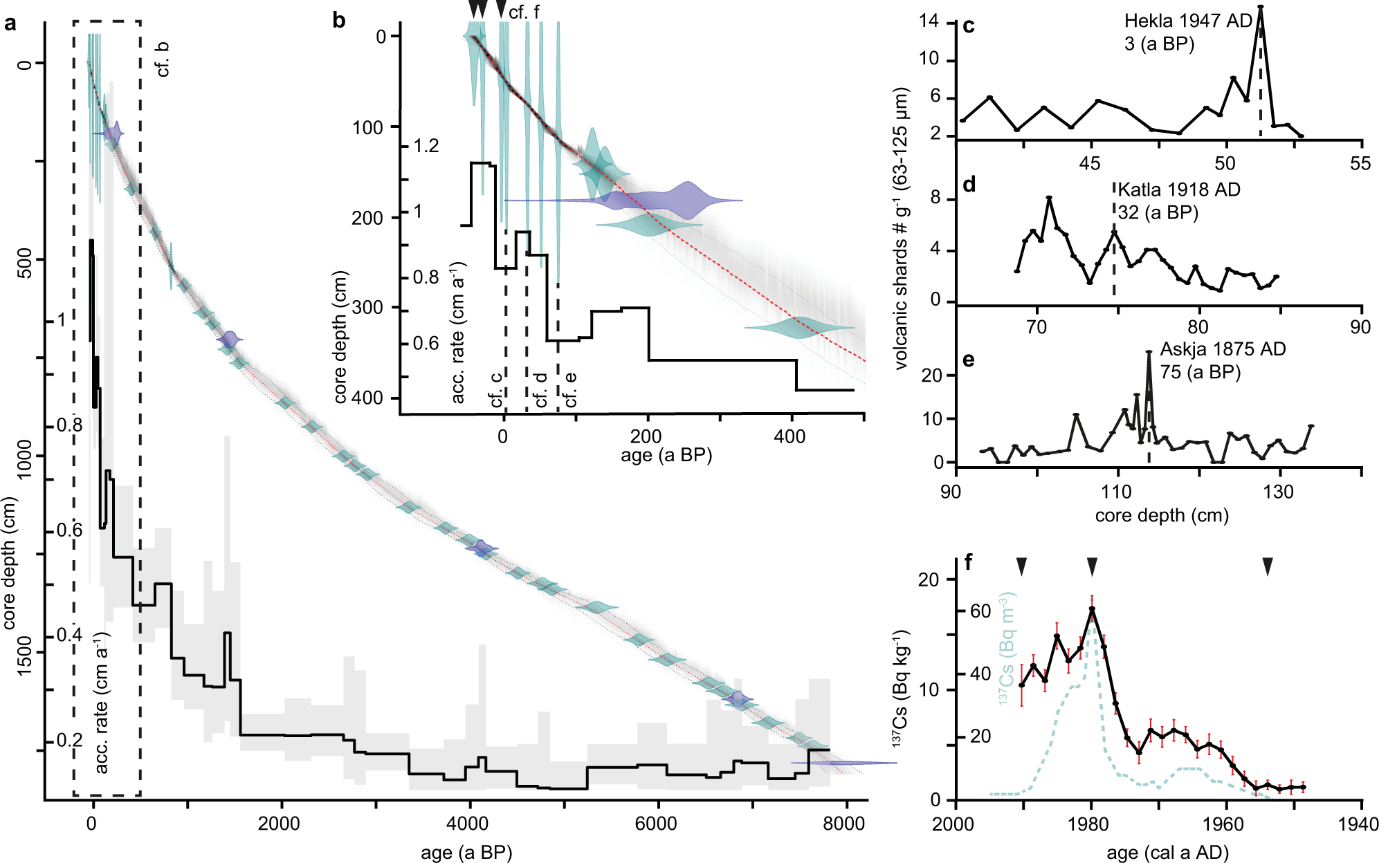


Fig. S1

**Age control, core GS13-182-01C** (after Becker et al. 2020). Sediment age was determined by correlation of the high resolution XRF records of Ca/Fe from core GS13 and from the previously well-dated record of nearby core P1-003 ^14,15^ along with new ^14^C dates, ^210^Pb/^137^Cs measurements, and tephra grain counts in GS13 itself. **a)** GS13 AMS ^14^C dates (purple), ^210^Pb/^137^Cs (red), tephra (triangles) and Ca/Fe tie-points (green) used to derive the age model. Age model uncertainties were determined from estimated uncertainties of individual age control points and a resulting ensemble of age model iterations. The grey shaded area denotes the 95% confidence interval around the median age model represented by the red line. The derived accumulation rate associated with the median age model is given by the black line along with its estimated 5–95% quantile range (grey area). **b)** Enlarged view of the last 550 years, with age control estimates from tephra counts (black triangles, per panels **c-e**) and ^137^Cs (panel **f)**. **c–e)** Tephra counts of basaltic-intermediate and rhyolitic shards in the 63–125 μm fraction denoting the number of shards related to three historic eruptions (for details see Becker *et al.^3^*). **f)** All ^137^Cs measurements from ^210^Pb/^137^Cs dating (n = 25) compared to the total ^137^Cs concentration in the SW Barents Sea ^16^. The triangles denote the ^137^Cs events used for age modelling. See **Supplementary Table S1** for age model input.


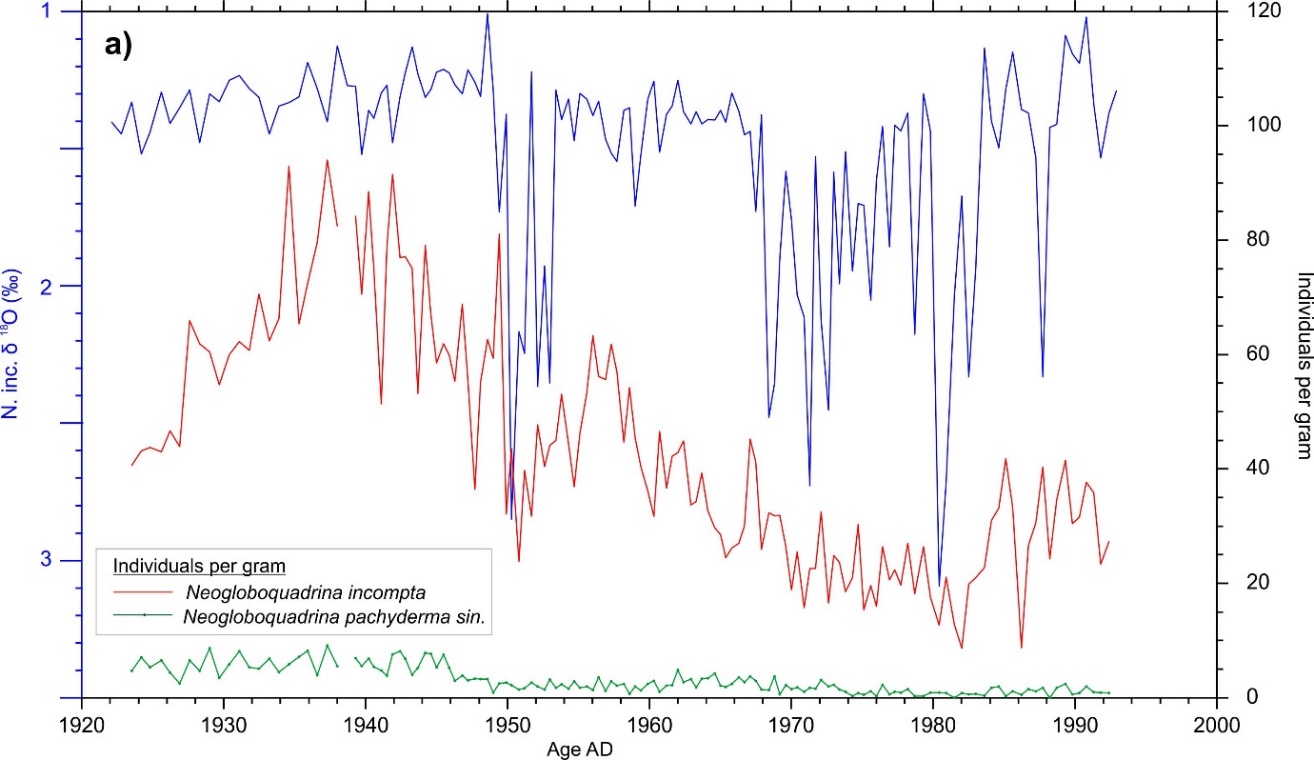


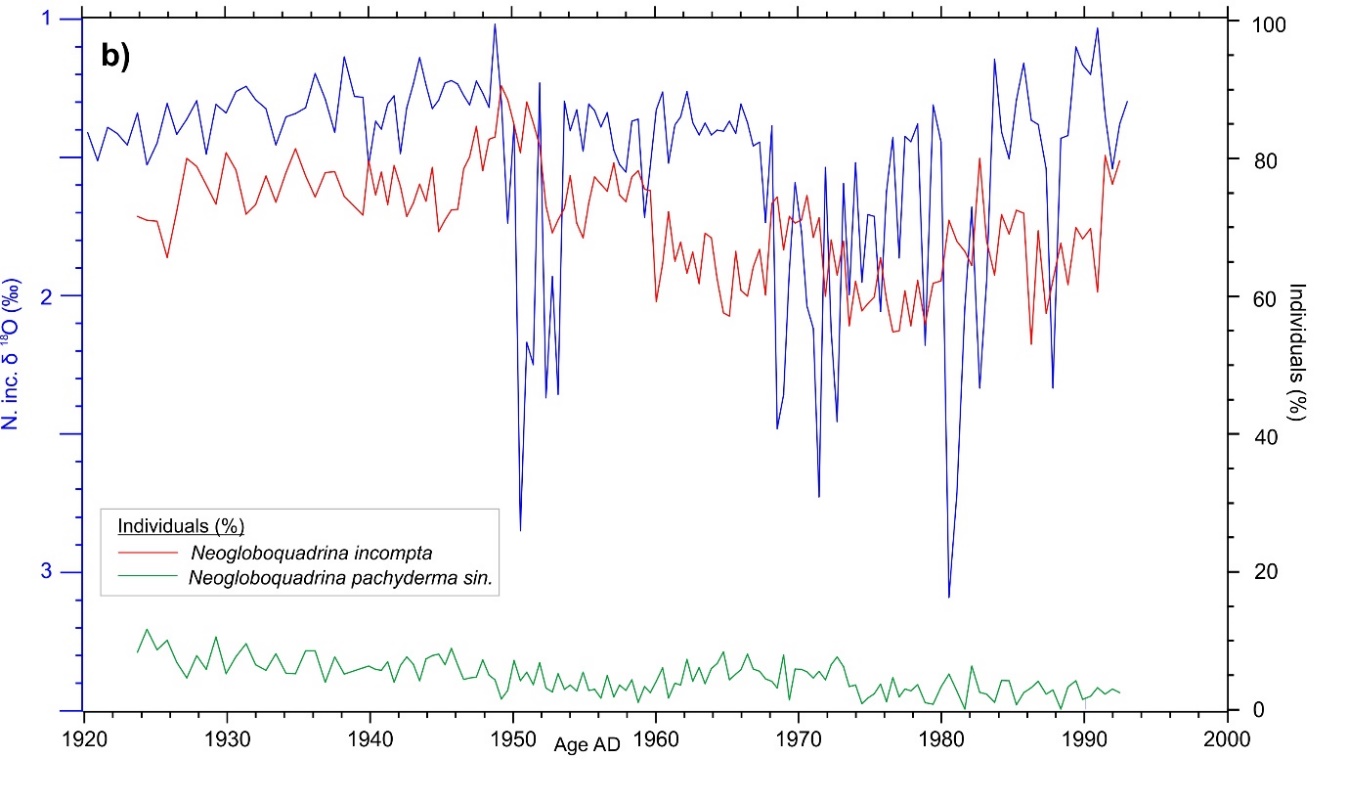


Fig. S2.

a) Absolute abundances of *Neogloboquadrina incompta* (red curve) and *Neogloboquadrina pachyderma sin*. (green curve) in the upper part of core GS13, expressed as number of individual specimens per gram of dry sediment, compared to d^18^O results (Fig. 2b; blue curve) discussed in the main text. b) As in a) but expressed as relative abundance of the same species in terms of percent fraction of the total planktonic foraminiferal fauna.


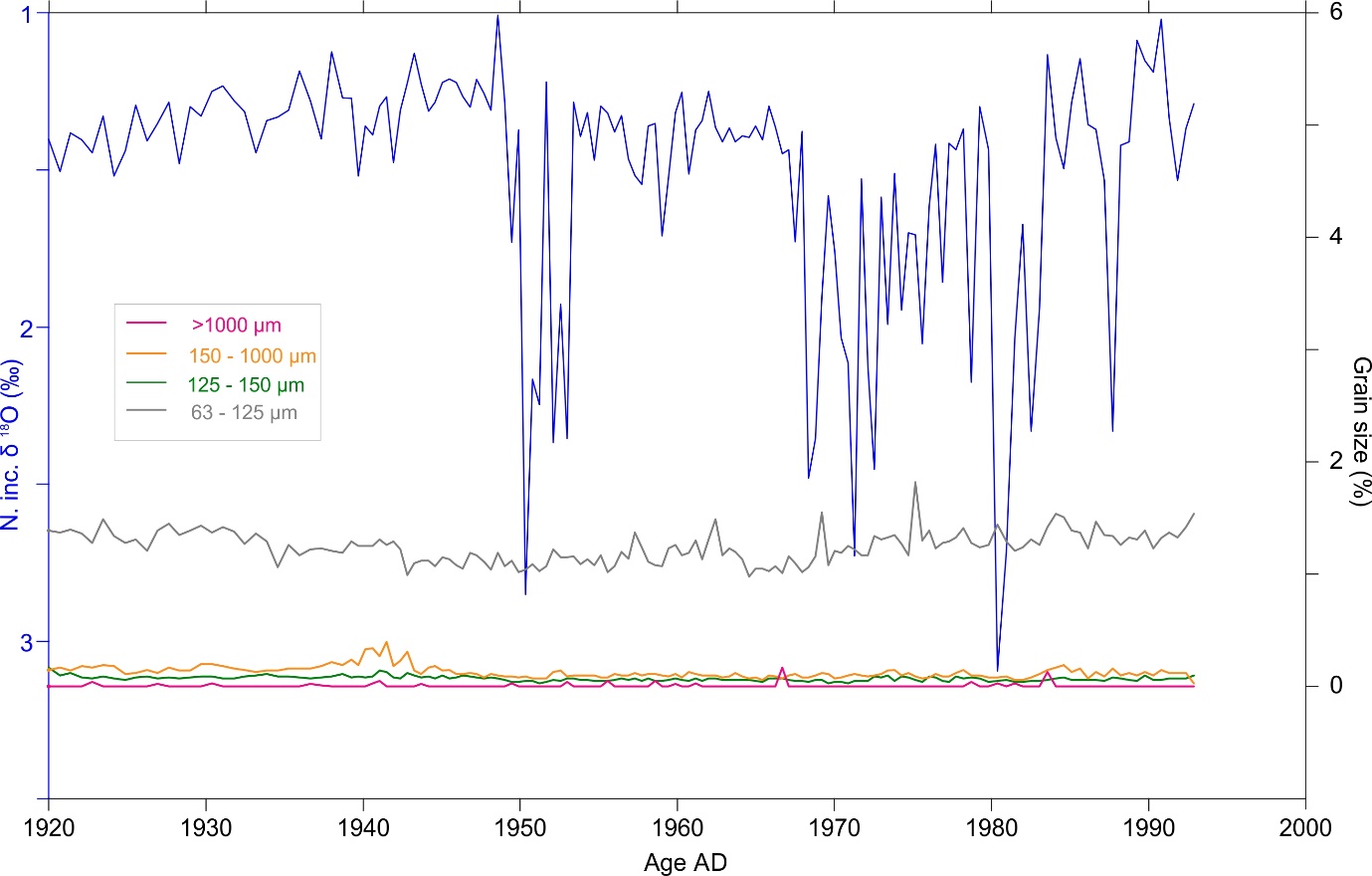


Fig. S3.

Grain size distribution of sediments in the upper part of core GS13 compared to d^18^O results (Fig. 2b) discussed in the main text.


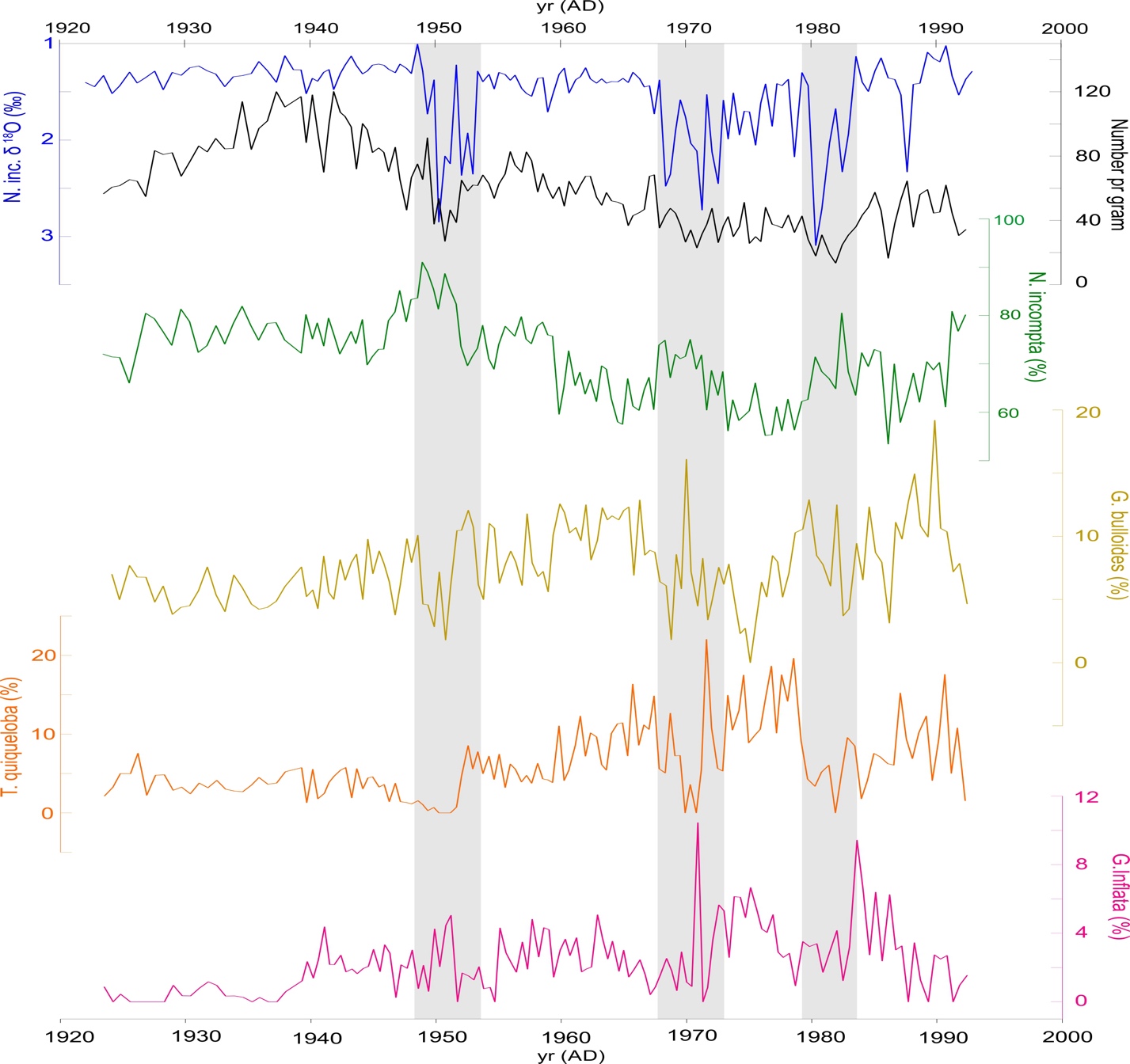


**Fig. S4.**

Planktonic foraminiferal biostratigraphy and oxygen isotope record in GS13 back to 1920 AD.


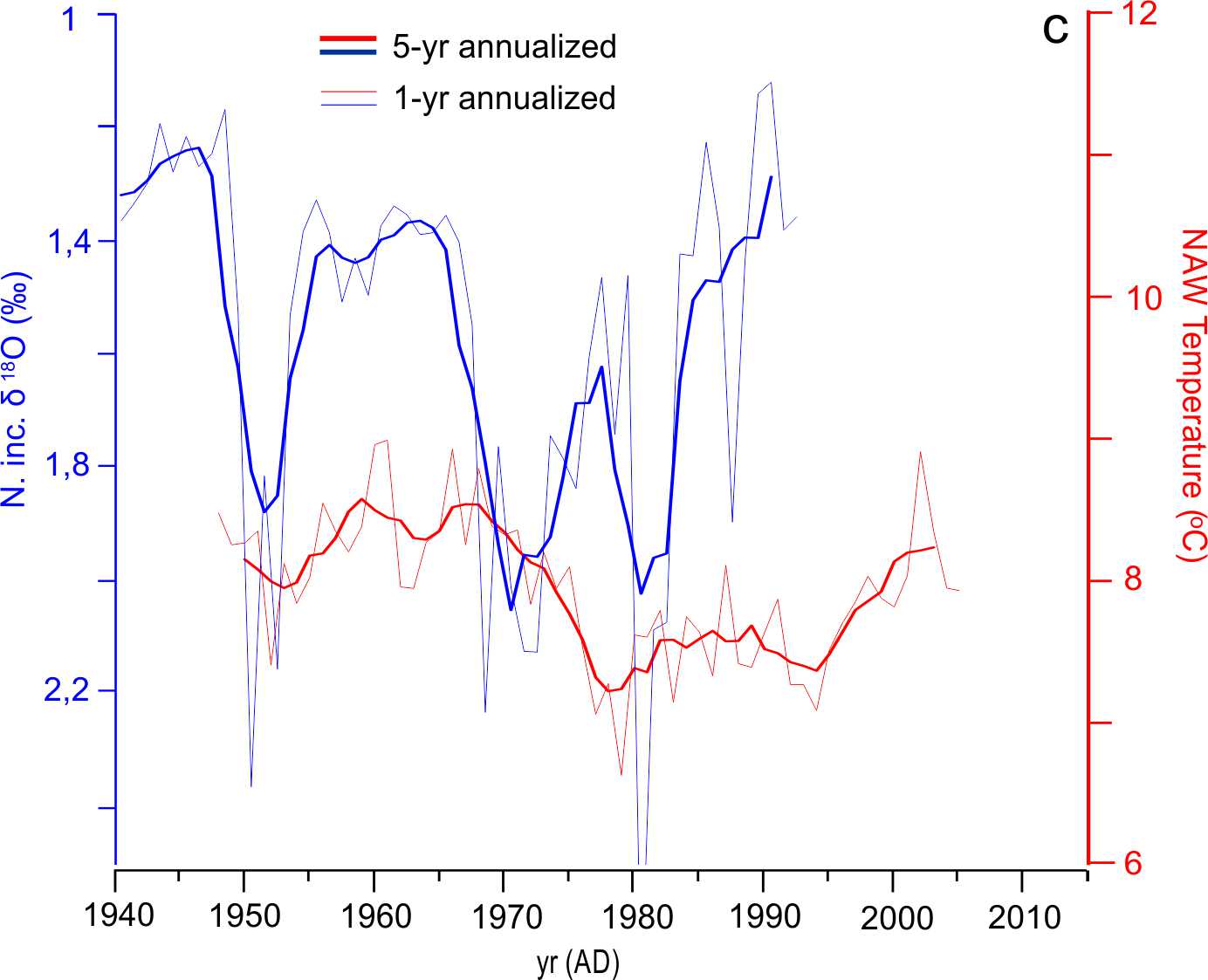


**Fig. S5.**

Annualized and five year annualized oxygen isotope data on *N. inc*. from core GS13 (blue) and temperature of inflow water (red) from the Faroe-Shetland region (NAW in Fig. 1)^17^.

Table S1.

Age model control ages for core GS13 (after Becker *et al.*^3^).

| ID | depth from (cm) | depth to (cm) | depth (cm) | 14C  age (a BP) | ± STD  (a) | !R (a) | ± !STD  (a) | !13C (‰) | age  (cal. a BP) | ± STD  (a) | median modelled age (cal. a BP) | ± 2 sigma STD (a) | material / type |
| --- | --- | --- | --- | --- | --- | --- | --- | --- | --- | --- | --- | --- | --- |
| 210Pb top of core |  |  | 0.00 |  |  |  |  |  | -42 | 3 | -43.6 | 15.7 | 137Cs event |
| 210Pb sellafield |  |  | 13.00 |  |  |  |  |  | -30 | 1 | -30.1 | 2.7 | 137Cs event |
| 210Pb first Cs |  |  | 43.00 |  |  |  |  |  | -4 | 1 | -4.1 | 2.5 | 137Cs event |
| Hekla1947 |  |  | 51.25 |  |  |  |  |  | 3 | 1 | 3.3 | 2.5 | tephra |
| Katla1918 |  |  | 75.25 |  |  |  |  |  | 32 | 1 | 31.9 | 3.0 | tephra |
| tie-point #1 |  |  | 93.00 |  |  |  |  |  | 51 | 1 | 51.0 | 2.9 | Ca/Fe tie-point |
| Askja1875 |  |  | 113.8 |  |  |  |  |  | 74.76 | 1 | 74.8 | 2.8 | Ca/Fe tie-point |
| tie-point #2 |  |  | 141.20 |  |  |  |  |  | 122 | 6 | 119.8 | 11.7 | Ca/Fe tie-point |
| tie-point #3 |  |  | 152.00 |  |  |  |  |  | 139 | 8 | 137.2 | 14.2 | Ca/Fe tie-point |
| CURL-19081 | 180.80 | 182.30 | 181.30 | 775 | 20 | 190 | 5 | 2.7 |  |  | 178.1 | 29.9 | N. incompta & G. bulloides |
| tie-point #4 |  |  | 208.30 |  |  |  |  |  | 201 | 20 | 216.6 | 34.1 | Ca/Fe tie-point |
| tie-point #5 |  |  | 321.60 |  |  |  |  |  | 409 | 21 | 421.2 | 43.8 | Ca/Fe tie-point |
| tie-point #6 |  |  | 430.35 |  |  |  |  |  | 661 | 17 | 656.3 | 37.5 | Ca/Fe tie-point |
| tie-point #7 |  |  | 517.65 |  |  |  |  |  | 829 | 6 | 829.8 | 18.5 | Ca/Fe tie-point |
| tie-point #8 |  |  | 566.55 |  |  |  |  |  | 968 | 21 | 964.7 | 41.1 | Ca/Fe tie-point |
| tie-point #9 |  |  | 636.15 |  |  |  |  |  | 1171 | 31 | 1175.6 | 51.4 | Ca/Fe tie-point |
| tie-point #10 |  |  | 663.50 |  |  |  |  |  | 1270 | 23 | 1264.1 | 45.9 | Ca/Fe tie-point |
| CURL-19083 | 703.10 | 705.10 | 703.60 | 2080 | 20 | 175 | 5 | 5.4 |  |  | 1394.4 | 54.2 | N. incompta & G. bulloides |
| tie-point #11 |  |  | 728.40 |  |  |  |  |  | 1425 | 28 | 1454.8 | 51.2 | Ca/Fe tie-point |
| tie-point #12 |  |  | 763.30 |  |  |  |  |  | 1551 | 29 | 1563.7 | 60.4 | Ca/Fe tie-point |
| tie-point #13 |  |  | 864.60 |  |  |  |  |  | 2039 | 31 | 2030.8 | 71.8 | Ca/Fe tie-point |
| tie-point #14 |  |  | 925.10 |  |  |  |  |  | 2324 | 32 | 2311.8 | 69.9 | Ca/Fe tie-point |
| tie-point #15 |  |  | 999.90 |  |  |  |  |  | 2658 | 34 | 2656.1 | 68.0 | Ca/Fe tie-point |
| tie-point #16 |  |  | 1023.95 |  |  |  |  |  | 2779 | 33 | 2772.4 | 62.6 | Ca/Fe tie-point |
| tie-point #17 |  |  | 1045.95 |  |  |  |  |  | 2912 | 35 | 2891.8 | 62.0 | Ca/Fe tie-point |
| tie-point #18 |  |  | 1129.35 |  |  |  |  |  | 3347 | 38 | 3352.6 | 93.2 | Ca/Fe tie-point |
| tie-point #19 |  |  | 1181.90 |  |  |  |  |  | 3741 | 36 | 3720.2 | 86.5 | Ca/Fe tie-point |
| tie-point #20 |  |  | 1212.25 |  |  |  |  |  | 3988 | 40 | 3950.4 | 79.2 | Ca/Fe tie-point |
| CURL-19126 | 1233.25 | 1234.25 | 1233.75 | 4255 | 20 | 175 | 5 | 0.8 |  |  | 4088.1 | 70.4 | N. incompta & G. bulloides |
| tie-point #21 |  |  | 1247.40 |  |  |  |  |  | 4166 | 35 | 4166.3 | 66.3 | Ca/Fe tie-point |
| tie-point #22 |  |  | 1296.15 |  |  |  |  |  | 4507 | 37 | 4496.1 | 87.9 | Ca/Fe tie-point |
| tie-point #23 |  |  | 1322.60 |  |  |  |  |  | 4764 | 46 | 4717.2 | 94.5 | Ca/Fe tie-point |
| tie-point #24 |  |  | 1337.55 |  |  |  |  |  | 4870 | 44 | 4845.5 | 83.2 | Ca/Fe tie-point |
| tie-point #25 |  |  | 1383.25 |  |  |  |  |  | 5343 | 64 | 5243.1 | 134.4 | Ca/Fe tie-point |
| tie-point #26 |  |  | 1465.75 |  |  |  |  |  | 5789 | 46 | 5774.3 | 104.0 | Ca/Fe tie-point |
| tie-point #27 |  |  | 1515.65 |  |  |  |  |  | 6088 | 57 | 6086.0 | 118.1 | Ca/Fe tie-point |
| tie-point #28 |  |  | 1574.95 |  |  |  |  |  | 6527 | 49 | 6505.1 | 117.0 | Ca/Fe tie-point |
| tie-point #29 |  |  | 1595.65 |  |  |  |  |  | 6685 | 53 | 6656.8 | 81.2 | Ca/Fe tie-point |
| CURL-19082 | 1615.65 | 1616.60 | 1616.15 | 6545 | 25 | 175 | 5 | 1 |  |  | 6797.0 | 73.3 | N. incompta & G. bulloides |
| tie-point #30 |  |  | 1630.25 |  |  |  |  |  | 6880 | 50 | 6881.1 | 72.0 | Ca/Fe tie-point |
| tie-point #31 |  |  | 1676.10 |  |  |  |  |  | 7160 | 49 | 7161.0 | 97.8 | Ca/Fe tie-point |
| tie-point #32 |  |  | 1714.65 |  |  |  |  |  | 7489 | 38 | 7448.6 | 92.2 | Ca/Fe tie-point |
| tie-point #33 |  |  | 1735.25 |  |  |  |  |  | 7635 | 45 | 7591.2 | 79.0 | Ca/Fe tie-point |
| Storegga +400 a (R) |  |  | 1777.90 | 7650 | 250 | 175 | 5 | NA |  |  | 7817.4 | 150.2 | Ref. 17 |

Table S2.

Stable isotope timeseries for core GS13 (see also: 10.5281/zenodo.10617999 (will be published))

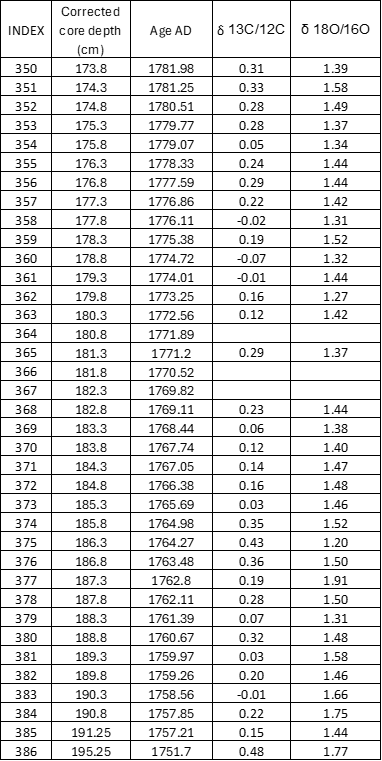


Table S3.

Replicate isotopic analysis included some levels with heavy isotope anomalies.

| **Age AD** | **d ^13^C/^12^C Run 1** | **d ^18^O/^16^O Run 1** | **d ^13^C/^12^C Run 2** | **d ^18^O/^16^O Run 2** | **d ^13^C/^12^C Run 3** | **d ^18^O/^16^O Run 3** | **n** | **d ^13^C/^12^C Average** | **d^18^O/^16^O Average** | **d^18^O/^16^O STDEV** |
| --- | --- | --- | --- | --- | --- | --- | --- | --- | --- | --- |
| 1987,70 | -0,14 | 2,33 | -0,44 | 2,04 | -0,35 | 4,09 | 3 | -0,31 | 2,82 | 1,11 |
| 1982,52 | 0,24 | 2,33 | 0,22 | 2,54 |  |  | 2 | 0,23 | 2,44 | 0,15 |
| 1980,94 | 0,11 | 2,71 | 0,06 | 3,17 | -0,46 | 2,81 | 3 | -0,10 | 2,90 | 0,24 |
| 1980,38 | 0,19 | 3,09 | 0,16 | 3,11 |  |  | 3 | 0,18 | 3,10 | 0,01 |
| 1978,72 | 0,23 | 2,18 | 0,16 | 1,83 | -0,13 | 1,74 | 3 | 0,09 | 1,91 | 0,23 |
| 1901,01 | 1,18 | 3,12 | 0,49 | 1,94 |  |  | 2 | 0,84 | 2,53 | 0,83 |
| 1900,46 | 0,34 | 1,43 | 0,28 | 1,42 |  |  | 2 | 0,31 | 1,43 | 0,01 |
| 1776,11 | -0,02 | 1,31 | 0,16 | 1,40 |  |  | 2 | 0,07 | 1,36 | 0,06 |

**Table S4.**

Reanalysis of *N. inc*. in size fractions 212-250 μm and 150-212 μm, through the first isotope anomaly.

**Table S5.**

Representative estimate of the development of hydrographic observations in the Nordic Seas, expressed as the number of hydrocasts (OSD plus XBT) from 60^o^-80^o^N x 40^o^W-20^o^E in 5 year bins available from the WODB09 (<https://www.nodc.noaa.gov/OC5/WOD09/pr_wod09.html> )

| **Year** | **No of casts** |
| --- | --- |
| 1930 | 2468 |
| 1935 | 3528 |
| 1940 | 5551 |
| 1945 | 4335 |
| 1950 | 8440 |
| 1955 | 20036 |
| 1960 | 24391 |
| 1965 | 23206 |

**References**

1 Becker, L. W. M., Sejrup, H. P., Hjelstuen, B. O., Haflidason, H. & Dokken, T. M. Ocean-ice sheet interaction along the SE Nordic Seas margin from 35 to 15 ka BP. *Marine Geology* (2017). <https://doi.org:10.1016/j.margeo.2017.09.003>

2 Klitgaard-Kristensen, D., Sejrup, H. P. & Haflidason, H. The last 18 kyr fluctuations in Norwegian Sea surface conditions and implications for the magnitude of climatic changes: Evidence from the North Sea. *Paleoceanography* **16**, 455-467 (2001).

3 Becker, L. W. M. *et al.* Palaeo-productivity record from Norwegian Sea enables North Atlantic Oscillation (NAO) reconstruction for the last 8000 years. *Npj Clim Atmos Sci* **3** (2020). <https://doi.org:ARTN> 42

10.1038/s41612-020-00147-6

4 Pearson, P. N. *et al.* Warm tropical sea surface temperatures in the Late Cretaceous and Eocene epochs (vol 413, pg 481, 2001). *Nature* **414**, 470-470 (2001). <https://doi.org:Doi> 10.1038/35106617

5 Osman, M. B. *et al.* Industrial-era decline in subarctic Atlantic productivity. *Nature* **569**, 551-+ (2019). <https://doi.org:10.1038/s41586-019-1181-8>

6 Boyce, D. G., Lewis, M. R. & Worm, B. Global phytoplankton decline over the past century. *Nature* **466**, 591-596 (2010). <https://doi.org:10.1038/nature09268>

7 Andersson, C., Risebrobakken, B., Jansen, E. & Dahl, S. O. Late Holocene surface ocean conditions of the Norwegian Sea (Voring Plateau). *Paleoceanography* **18** (2003). <https://doi.org:Artn> 1044

10.1029/2001pa000654

8 Pflaumann, U., Duprat, J., Pujol, C. & Labeyrie, L. D. SIMMAX: A modern analog technique to deduce Atlantic sea surface temperature from planktonic foraminifera in deep-sea sediments. *Paleoceanography* **11**, 15-35 (1996).

9 Risebrobakken, B., Jansen, E., Andersson, C., Mjelde, E. & Hevrøy, K. A high-resolution study of Holocene paleoclimatic and paleoceanographic changes in the Nordic Seas. *Paleoceanography* **18**, 1017 (2003).

10 Husum, K. & Hald, M. Arctic planktic foraminiferal assemblages: Implications for subsurface temperature reconstructions. *Mar Micropaleontol* **96-97**, 38-47 (2012). <https://doi.org:10.1016/j.marmicro.2012.07.001>

11 Jonkers, L. & Kucera, M. Global analysis of seasonality in the shell flux of extant planktonic Foraminifera. *Biogeosciences* **12**, 2207-2226 (2015). <https://doi.org:10.5194/bg-12-2207-2015>

12 Spooner, P. T. *et al.* Exceptional 20th Century Ocean Circulation in the Northeast Atlantic. *Geophys Res Lett* **47** (2020). <https://doi.org:ARTN> e2020GL087577

10.1029/2020GL087577

13 Sahoo, N. *et al.* Planktic Foraminiferal Assemblages in Surface Sediments From the Subpolar North Atlantic Ocean. *Front Mar Sci* **08** (2022). <https://doi.org:ARTN> 781675

10.3389/fmars.2021.781675

14 Sejrup, H. P., Haflidason, H. & Andrews, J. T. A Holocene North Atlantic SST record and regional climate variability. *Quat. Sci. Rev.* **30**, 3181-3195 (2011). <https://doi.org:Doi> 10.1016/J.Quascirev.2011.07.025

15 Sejrup, H. P. *et al.* Response of Norwegian Sea temperature to solar forcing since 1000 AD. *J Geophys Res-Oceans* **115** (2010). <https://doi.org:Artn> C12034

Doi 10.1029/2010jc006264

16 Gao, Y. Q., Drange, H., Bentsen, M. & Johannessen, O. M. Tracer-derived transit time of the waters in the eastern Nordic Seas. *Tellus B* **57**, 332-340 (2005). <https://doi.org:DOI> 10.1111/j.1600-0889.2005.00153.x

17 Eldevik, T. *et al.* Observed sources and variability of Nordic seas overflow. *Nat Geosci* **2**, 405-409 (2009). <https://doi.org:10.1038/Ngeo518>
